# Current practices of cytoreductive surgery and hyperthermic intraperitoneal chemotherapy in the treatment of peritoneal surface malignancies: an international survey of oncologic surgeons

**DOI:** 10.1186/s12957-018-1377-7

**Published:** 2018-05-15

**Authors:** Heon Jong Yoo, Jenny J. Hong, Young Bok Ko, Mina Lee, Youjin Kim, Hye Young Han, Yong Jung Song, Myong Cheol Lim, Sang-Yoon Park

**Affiliations:** 1 Department of Obstetrics and Gynecology, Chungnam National University College of Medicine, 33, Munhwa-ro, Jung-gu, Deajeon, 301-721 South Korea; 2 Department of Obstetrics and Gynecology, Chungnam National University Hospital, 33, Munhwa-ro, Jung-gu, Deajeon, 301-721 South Korea; 3 Surgical Oncology, Department of Surgery, MedStar Washington Hospital Center, 110 Irving St Suite 1E Room 1223, Washington, DC 20010 USA; 4 Department of Pediatrics, Chungnam National University College of Medicine, Daejon, South Korea; 5 Pusan National University Yangsan Hospital, Pusan National University School of Medicine, Pusan, South Korea; 6 Department of Obstetrics and Gynecology, Pusan National University Yangsan Hospital, Pusan National University School of Medicine, 20 Geumo-ro, Mulgeum-eup, Yangsan, 50612 South Korea; 7 Center for Uterine Cancer, Research Institute and Hospital, National Cancer Center, 323, Ilsan-ro, Ilsandong-gu, Goyang-si, Gyeonggi-do 410-769 South Korea

**Keywords:** Peritoneal surface malignancies, Cytoreductive surgery, Intraoperative hyperthermic intraperitoneal chemotherapy, International survey

## Abstract

**Background:**

The goal of the study was to investigate the current clinical practices among oncologic surgeons regarding cytoreductive surgery (CRS) with intraoperative hyperthermic intraperitoneal chemotherapy (HIPEC).

**Methods:**

From September to October 2016, an online questionnaire surveyed the oncologic surgeons by email. The questionnaire included 20 multiple-choice questions of the following: eligibility for the CRS with HIPEC procedure, perioperative staging and surgery skill, assessment of residual tumors, and method used for intraperitoneal HIPEC.

**Results:**

The response rate was 16% (34/217). The majority of respondents (68%) worked at a university hospital. All respondents indicated that mesenteric invasion is the most crucial factor affecting treatment decision. Most surgeons (79%) used the Sugarbaker’s staging system to intraoperatively measure the extent of peritoneal invasion. The methods used to measure the extent of miliary pattern of residual tumor spread, and the amount of residual tumor after electrocauterization varied among the surgeons. Most responders (65%) used the closed system of HIPEC.

**Conclusions:**

Despite the fact that CRS HIPEC is the standard treatment for PSM, the clinical practices are very different according to each clinical situation. Nevertheless, mesenteric invasion was found to be the most important factor impacting the treatment decision-making by the majority of responders.

**Electronic supplementary material:**

The online version of this article (10.1186/s12957-018-1377-7) contains supplementary material, which is available to authorized users.

## Background

Peritoneal surface malignancies (PSM) represent an advanced form of abdominal malignancies associated with a dismal prognosis which including gastric, colorectal, ovarian, peritoneal mesothelioma, and pseudomyxoma peritonei. Many patients have been treated with palliative surgical debulking procedures followed by systemic chemotherapy only [1]. However, the success of this treatment modality has been limited by peritoneal penetration [2]. Although PSM is categorized as a metastatic peritoneal disease, it may represent a special locoregional disease pattern limited to the abdominal cavity. Cytoreductive surgery (CRS) and intraoperative hyperthermic intraperitoneal chemotherapy (HIPEC) have been used as the standard locoregional treatment for selected patients with PSM [3, 4]. In well-selected patients with an acceptable disease burden, the multimodal modality of CRS with HIPEC may offer an extended survival compared to standard systemic chemotherapy or no treatment [5, 6]. However, given the perceived and well-recognized morbidities, the lack of widespread acceptance, adoption, and accessibility associated with CRS/HIPEC remain a significant challenge, even in recent inquiries [7, 8].

A few guidelines have been created to optimize the benefits and minimize the morbidity for patients with PSM [9, 10]. However, the treatments for PSM are complex and heterogeneous and require institutional support [11]. Therefore, actual practice protocols vary according to each surgeon’s preference despite general guidelines established from consensus obtained through a Delphi process. The standardization of clinical practice guidelines is paramount in improving patient selection, perioperative morbidity, and completeness of cytoreduction [12, 13].

The goal of this study was to investigate the variations in clinical practice of CRS with HIPEC among oncologic surgeons from different countries and to assess their opinions surrounding this therapeutic modality.

## Methods

From September to October 2016, an online questionnaire surveyed by the corresponding authors from various countries from published manuscripts on CRS with HIPEC and to the members of the Korean Society of PSM (KSPSM). The questionnaire was received from only one respondent per team. An English-language survey was developed by the authors and was made available online for anonymous submission using a web-based survey service (SurveyMonkey (R), Palo Alto, CA, USA). The full survey is available in Additional file 1. Surgeons were requested by email to complete the survey within a 4-week period, and approval was obtained from the institutional review board of the primary site (CNUH 2017-01-034-002). The questionnaire included 20 multiple-choice questions to assess the opinions of the responders; their criteria for patient selection; and eligibility for CRS with HIPEC, modes of perioperative staging, level of surgery skills, residual tumor assessment, and method used for HIPEC administration. A reminder was sent to non-respondents 3 to 4 weeks following the initial mailing of the questionnaire. The closing date of the survey was October 2016, and the results were analyzed. A descriptive analysis was performed. A subgroup analysis of the experience of CRS/HIPEC and hospital volume of the responders was performed using the *T* test for continuous variables (or the Kolmogorov-Smirnov test when the expected frequency within any cell was less than 5) and the *χ*^2^—test (or Fisher’s exact test when the expected frequency within any cell was less than 5) for the categorical variables.

## Results

### Demographics

The response rate was 16% (34/217), and the demographics of the respondents are detailed in Table 1. The majority of the respondents worked at a university hospital (68%), and the remainder worked at a cancer center (26%) and general hospital (6%). All respondents were oncologic surgeons. Respondents from 11 nationalities participated in this survey including general surgeons (79%) and gynecologic oncologists (21%). The general surgeons represented various subspecialties including colorectal (44%), gastric (24%), and hepatobiliary sections (3%). Most surgeons were older than 40 years of age (82%), and approximately half of the surgeons stated their volume to be higher than 30 cases. The indications for CRS/HIPEC included pseudomyxoma, colorectal cancer, ovarian cancer, gastric cancer, and peritoneal mesothelioma.

**Table 1:** Demographic characteristics of survey respondents

Volume of medical institution	*N* (%)
University hospital	23 (68%)
Cancer center	9 (26%)
General hospital	2 (6%)
Country	*N* (%)
Korea	16 (47%)
USA	3 (9%)
Italy	3 (9%)
France	2 (6%)
India	2 (6%)
Greece	2 (6%)
Japan	1 (3%)
Germany	1 (3%)
Netherlands	1 (3%)
Spain	1 (3%)
Canada	1 (3%)
Specialty	*N* (%)
General surgery (colorectal cancer)	15 (44%)
General surgery (gastric cancer)	8 (24%)
Obstetrics and gynecology (gynecologic cancer)	7 (21%)
General surgery (liver cancer)	1 (3%)
Age	
30s	7 (21%)
40s	16 (47%)
50s	4 (12%)
60s	7 (21%)
Experience number of PSM	*N* (%)
< 10	7 (21)
11–30	10 (29)
31–50	3 (9)
> 51	14 (41)
Major causes of PSM	*N* (%)
Pseudomyxoma	9 (24)
Ovarian cancer	9 (24)
Colorectal origin cancer	9 (24)
Gastric origin cancer	4 (11%)
Peritoneal mesothelioma	1 (3%)
Others	5 (14%)

### Eligibility for the CRS and HIPEC procedure

There was a unanimous agreement by the responders that diffuse mesenteric invasion is the most crucial factor affecting the selection of treatment options in peritoneal surface malignancies. Other factors impacting the treatment decision to pursue CRS/HIPEC were a poor Eastern Cooperative Oncology Group (ECOG) performance status (53%) followed by portal vein invasion (47%). Fewer than 20% of respondents stated that old age of patients, frozen pelvis, and urethral stricture were significant factors in their decision making to offer CRS/HIPEC. The surgeons with a lower case volume (less than 50 cases) had a higher propensity to take into account more clinical factors in their treatment decision compared to the higher volume surgeons (greater than 50 cases) (*P* = 0.079). The age of the respondents and the case volume of the corresponding medical institution were not associated with the number of clinical factors (Table 2).

**Table 2:** Eligibility to the procedure of CRS with HIPEC

Factors that interrupt the right treatment of CRS with HIPEC	*N* (%)	
Old age	7 (21)	
ECOG performance status	18 (53)	
Invasion to numerous mesenteries	34 (100)	
Cancer that invade multiple organs (more than 3 organs)	5 (15)	
Cancer that invade more than 3 parts of liver parenchyma	10 (29)	
Cancer that invade the portal vein	16 (47)	
Cancer that invade a frozen pelvis	7 (21)	
Ureteral stricture	2 (6)	
Others	2 (6)	
The number of factors that interrupt the right treatment of CRS with HIPEC	Mean (SD)	*P*
Surgeon’s age (*n*)		
< 50 (23)	2.41 ± 1.43	
> 50 (11)	2.81 ± 1.60	0.352
Volume of medical institution (*n*)		
University hospital (23)	2.42 ± 1.43	
Other hospital (11)	2.81 ± 1.60	0.488
Experience number of PSM (*n*)		
< 50 (20)	2.26 ± 1.49	
< 50 (14)	3.21 ± 1.31	0.079

Ninety-one percent of responders used CT for preoperative assessment of disease while the remainder of the surgeons preferred a whole-body PET/CT or MRI (50 vs 19%, respectively).

### Perioperative staging and surgery skill

Most surgeons used the Sugarbaker’s staging system [3] to intraoperatively measure the extent of peritoneal invasion. Others (21%) used their own peritoneal carcinomatous index (Table 3). A small number of surgeons used either written description (6%) or intraoperative photography (9%) to document the location and size of the tumors.

**Table 3:** Perioperative staging and assessment of residual tumor

During operation, how to measure the extent of peritoneal invasion?	*N* (%)
Sugarbaker’s staging	20 (59)
Own peritoneal carcinomatosis index	7 (21)
Expressing location and size of intra-abdominal tumor using descriptive method	2 (6)
Keeping pictures of each parts of abdomen	3 (9)
Others	
How to measure the size of residual tumor that spreads in a miliary shape but sticks together (conglomerate)?	*N* (%)
Measure the size of whole clustered group as one tumor	21 (62)
Measure the size of each small miliary-shaped tumor	9 (26)
Others	2 (6)
How to assess residual tumor?	
Measure the longest section of the residual tumor with naked eyes	28 (82)
Measure the shortest section of the residual tumor with naked eyes	1 (3)
Measure the longest section of the residual tumor with ruler	2 (6)
Measure the shortest section of the residual tumor with ruler	0
Measure the residual tumor with a ruler after taking an image	1 (3)
Others	
After performing not en bloc resection but electrocauterization in the area of miliary tumor spread, how do you assess residual tumor?	*N* (%)
If the residual tumor is not seen, define it as R0	16 (47)
If the residual tumor is not seen but there is leftover, define it as R1	13 (38)
Others	3 (9)

To measure the conglomerate area of miliary tumor spread, 62% of the responders measured the size of the whole group as one tumor cluster, while others (26%) measured the size of each small miliary-shaped tumor (Table 3). Most surgeons (82%) grossly assessed the amount of residual tumor according to the longest diameter of the residual tumor while few surgeons (6%) measured the longest diameter of the residual tumor area with a ruler. One surgeon (3%) used the shortest section of the residual tumor to assess the residual tumor with naked eyes. One respondent (3%) took an image of the residual tumor and measured it with a ruler after surgery.

Forty-seven percent of the respondents stated that if the residual tumor was not seen, they defined it as R0 after performing not en bloc resection but electrocauterization in the area of miliary tumor spread [14]. Thirty-eight of the respondents classified gross microscopic disease as R1 [14].

### Method for intra-abdominal HIPEC

Sixty-five percent of the respondents indicated that they administered HIPEC using a closed system (Table 4). Most surgeons (82%) used an FDA-authorized or commercially available perfusion machine for HIPEC. One surgeon (6%) used a self-made machine. More than half of the surgeons indicated that the infusion temperature of the intraperitoneal chemotherapy solution(s) was set to 42 °C (53%). Eighteen percent of the surgeons used a narrow temperature range between 41 and 43 °C. One surgeon perfused the solution using temperatures greater than 43 °C. Forty-eight percent of the respondents (13/27) used 90 min for total perfusion time while 30% surgeons (8/27) used 60 min. Nineteen percent of the respondents (5/27) reported a 30-min perfusion period. Only one surgeon used 120 min. The cost it takes to perform HIPEC, excluding the price of running the machine and chemotherapy agents, was above $4000 for 42% of the surgeons (11/26), $1000–2000 for 31% (8/26), and about $2000–$3000 for 19% of the responders (5/26). Few respondents indicated that the price of HIPEC was below $1000 (2/26).

**Table 4:** Method of intra-abdominal HIPEC

The method of intra-abdominal HIPEC	*N* (%)
HIPEC with open method	8 (31)
HIPEC with closed method	17 (65)
Others	1 (4)
Type of HIPEC machine	*N* (%)
Use FDA-authorized machine or commercially available machine	23 (85)
Use self-produced machine	2 (8)
Others	2 (7)
The temperature of infusing liquid while performing HIPEC	*N* (%)
Under 40°°C	0 (0)
40°°C	1 (4)
41°°C	6 (21)
42°°C	14 (50)
43°°C	6 (21)
Above 43°°C	1 (4)
HIPEC perfusion time	*N* (%)
60 min	8 (30)
90 min	13 (48)
120 min	1 (4)
Others	5 (18)<- 19
Performing cost of HIPEC except the price of running a machine and chemotherapy agents	*N* (%)
About $1000	2 (8)
$1000–$2000	8 (31)
$2000–$3000	5 (19) <-24
$3000–$4000	0 (0)
$4000	11 (42) <-52

Among the respondents who selected HIPEC using a closed method, 47% (9/19) of the surgeons sutured the skin prior to HIPEC infusion and then re-opened the abdomen for final evaluation and closure. Thirty-seven percent of the respondents (7/19) sutured the skin and performed HIPEC followed by removal of the perfusion tubes and drains. Fewer surgeons (2/19) sutured the fascia prior to HIPEC and then sutured the skin followed by removal of the perfusion tubes. One surgeon sutured the fascia and performed HIPEC, and then re-opened the abdomen for assessment prior to final closure.

## Discussion

Our study indicated that diffuse mesenteric invasion is the most crucial factor impacting the treatment decision for PSM. Approximately half of the respondents indicated that a poor ECOG performance status and portal vein invasion were the second and third factors, respectively, as challenges against the use of CRS with HIPEC. Previous studies have reported that high-volume surgeons could produce favorable oncologic outcomes and reduce adverse events [13, 15, 16]. The present study showed that surgeons who have performed at least 50 cytoreductive surgeries encountered fewer obstacles perioperatively and morbidities, and revealed that the volume of the medical institutions may not be the critical factor.

There was no doubt that the number of experienced CRS/HIPEC by the surgeons was one of the important factors for the treatment outcomes of PSM. However, Gary et al. reported that even low-volume experiences with CRS/HIPEC can lead to a reduction in adverse perioperative events with acceptable oncologic outcomes [17]. In addition, Cliby et al. reported that CRS was greatly influenced by surgical effort, and the rate of optimal cytoreduction was depended upon surgeon tendency [18], which implies that the individual surgeon’s skills and effort may be as important as the experiences of the surgeon in the oncologic outcomes of CRS. In order to achieve the optimal outcome, all surgeons that perform CRS/HIPEC were expected to have received proper training and asked to strictly follow the guidelines.

CRS and HIPEC have been shown to improve survival in selected patients with PSM, and it can be considered as the standard of care for this specific disease entity [19, 20]. Despite its widespread use in clinical practice, CRS with HIPEC continues to be perceived by a sizable proportion of the surgical community as experimental, possibly related to a lack of inclusion in clinical practice guidelines [9]. In this study, most surgeons used an FDA-authorized or a commercially available machine during HIPEC. However, current practices regarding infusion temperature during HIPEC have widely varied. While half of the surgeons used 42°, *t* temperatures ranged from 41, 43, 40, and over 43 °C (18, 18, 6, and 6%, respectively). The HIPEC perfusion time is also different, with approximately half of the surgeons indicating 90 min while others preferred a treatment time of 60, 30, and 120 min (48, 30, and 19%, respectively). Likewise, inconsistencies in the various technical practice patterns may significantly impact the clinical outcomes. Despite the complexity of PSM, a standardization of practice protocols must be formulated.

We demonstrated that most surgeons used the Sugarbaker’s staging system to intraoperatively measure the extent of peritoneal invasion. However, a few others use their own non-standardized peritoneal carcinomatous index. Furthermore, clinicians are using different methods to measure the residual tumor size. Most responders measured the size of the entire clustered group as one tumor while others individually measured the size of each tumor nodule. The methods to standardize perioperative staging are paramount.

Although the originally described closed technique is limited by a lack of direct control to the abdominal cavity, resulting in a suboptimal homogeneous distribution of the perfusate, it may provide the highest level of protection for the operating room staff. In this study, almost all respondents used the closed method. However, there is also no standard protocol described for HIPEC using the closed method. Approximately half of the surgeons who selected HIPEC with a closed method first sutured the skin closed, performed HIPEC, followed by re-opening of the abdomen for final assessment and closure. Other methods used include various permutations of the closed method. Further studies are needed to evaluate which strategy of the closed method would maximize clinical outcome, efficiency, and safety for the operating staff.

The limitations of this study include the potential for self-reported bias. The sample size was small and exhibited heterogeneity. Our results should be further verified in future studies using a large prospective cohort. Despite these limitations, this study warrants the importance of further large-scale studies to evaluate and subsequently recommend clinical guidelines for CRS with HIPEC in the treatment of PSM. Although CRS with HIPEC is currently the recommended treatment for PSM, practices vary widely. Therefore, the standardization of clinical practice protocols will provide an opportunity to improve patient outcomes.

## Conclusion

The CRS and HIPEC have been used as the standard treatment for selected patients with PSM. However, the clinical practices of CRS with HIPEC are very different according to each clinical situation. Nevertheless, mesenteric invasion was found to be the most important factor impacting the treatment decision-making by the majority of responders.

## Additional file

Additional file 1: Survey about treating patient with peritoneal surface malignancy. (DOC 35 kb)
