# Supplementary material for: Current practices of cytoreductive surgery and hyperthermic intraperitoneal chemotherapy in the treatment of peritoneal surface malignancies: an international survey of oncologic surgeons
Source: World J Surg Oncol. 2018 May 15;16:92. doi: 10.1186/s12957-018-1377-7 (PMC5952844; doi:10.1186/s12957-018-1377-7)
Supplement: Supplementary file 1 — Survey about treating patient with peritoneal surface malignancy. (DOC 35 kb) [file 12957_2018_1377_MOESM1_ESM.doc]

This survey is conducted by korean society of peritoneal surface malignancies and the goal of this survey is to understand the treatment pattern regarding treatment of patient with peritoneal surface malignancy. If you fill out this survey the way you treat in real life, it could only be used as a good reference treating these patient. You won't get any penalty from this survey and the patient information you offer will be protected.

Survey about treating patient with peritoneal surface malignancy

**I. Analysis of people who join this survey**

**1. What kind of a medical institute do you work at?**

1. University hospital 2. Cancer center 3. General hospital 4. Clinic

**2. What country do you work at?**

_________________________

**3. What is your specialty?**

1. General surgery(gastric cancer)

2. General surgery(colorectal cancer)

3. General surgery(liver cancer)

4. General surgery(lung cancer)

5. Obstetrics and gynecology(gynecologic cancer)

**4. What is your age?**

1. Thirties 2. Forties 3. Fifties 4. Sixties 5. Above seventy

**5. How many surgery of peritoneal surface malignancies are done in your hospital?**

1. None 2. Below 10 3. 11~30 4. 30~50 5. Above 50

**6. How many surgery of peritoneal surface malignancies do you perform?**

1. None 2. Below 10 3. 11~30 4. 30~50 5. Above 50

**7. What is a major cause of surgery of peritoneal surface malignancies in your hospital?**

1. Pseudomyxoma

2. Peritoneal mesothelioma

3. Ovarian cancer

4. Gastric origin cancer

5. Colorectal origin cancer

6. Others

**II. Eligibility to the procedure of CRS and/or HIPEC)**

**8. What is/are factors that interrupt you choosing right treatment of peritoneal surface malignancies? (you can choose multiple answers)**

1. Old age

2. ECOG performance status

3. Invasion to numerous mesenteries

4. Cancer that invades multiorgans(more than 3 organs)

5. Cancer that invades more than 3 parts of liver parenchyma

6. Cancer that invades portal vein

7. Cancer that invades frozen pelvis

8. Ureteral stricture

9. Others

**9. Before surgery, what diagnostic imaging device do you use for measuring extent of cancer? (you can choose multiple answers)**

1. Abdominal ultrasonography

2. Abdominal CT

3. Abdominal MRI

4. Whole body PET CT

5. Others

**III. Perioperative staging and surgery skill**

**10. During operation, what kind of method do you use to measure the extent of peritoneal invasion?**

1. Sugarbaker's staging

2. Own PCI (Peritoneal carcinomatosis index)

3. Expressing location and size of intra-abdominal tumor using technical method

4. Keeping pictures of each parts of abdomen

5. Others

**11. How do you measure the size of residual tumor that spreads in a miliary shape but sticks together(conglomerate)?**

1. Measure the size of whole clustered group as one tumor

2. Measure the size of each small miliary shaped tumor

3. Others

**IV. Assessment of residual tumor**

**12. How do you assess residual tumor?**

1. Measure the longest section of residual tumor with naked eyes

2. Measure the shortest section of residual tumor with naked eyes

3. Measure the longest section of residual tumor with ruler

4. Measure the shortest section of residual tumor with ruler

5. After taking image of residual tumor and measure it with ruler

6. Others

**13. After performing electrocauterization not en bloc resection, how do you assess residual tumor?**

1. If the residual tumor is not seen, define it as Ro.

2. If the residual tumor is not seen but there is leftover, define it as R1.

3. Others

**V. Method of intra-abdominal HIPEC**

**14. Question about the method of intra-abdominal HIPEC**

1. HIPEC with open method

2. HIPEC with closed method

3. Others

**15. Question about the HIPEC machine**

1. Use FDA authorized machine or commercialized machine

2. Use self-produced machine

3. Others

**16. Question about the temperature of infusing liquid while performing HIPEC**

1. Under 40 degree celcius

2. 40 degree celcius

3. 41 degree celcius

4. 42 degree celcius

5. 43 degree celcius

6. Above 43 degree celcius

**17. Question about chemotherapy agents while performing HIPEC (In question #5, it is the drug used to treat peritoneal surface malinancies)**

1. Mitomycin-C

2. Oxaliplatin

3. 5 FU

4. Cisplatine

5. Carboplatine

6. Taxol

7. Others

**18. The procedure of HIPEC (only who selected HIPEC with closed method in question #14)**

1. After suturing skin and performing HIPEC, remove perfusion tube

2. After suturing fascia, performing HIPEC, suture skin and remove perfusion tube

3. After suturing skin and performing HIPEC, open the abdomen and confirm that everything is done well

4. After suturing fascia and performing HIPEC, open the abdomen and confirm that everything is done well

**19. How long is HIPEC perfusion time?**

1. 60 minutes

2. 90 minutes

3. 120 minutes

4. Others

**20. Question about the cost it takes performing HIPEC except the price of running a machine and chemotherapy agents**

1. Under 1,000,000 won(about $1,000)

2. 1,000,000~2,000,000 won(about $1,000~$2,000)

3. 2,000,000~3,000,000 won(about $2,000~$3,000)

4. 3,000,000~4,000,000 won(about $3,000~$4,000)

5. Above 4,000,000 won(about $4,000)

Thank you for your participation

Korean society of peritoneal surface malignancies
